# Use of Machine Learning and Routine Laboratory Tests for Diabetes Mellitus Screening

**DOI:** 10.1155/2022/8114049

**Published:** 2022-03-29

**Authors:** Glauco Cardozo, Guilherme Brasil Pintarelli, Guilherme Rettore Andreis, Annelise Correa Wengerkievicz Lopes, Jefferson Luiz Brum Marques

**Affiliations:** ^1^Academic Department of Health and Services, Federal Institute of Santa Catarina, Florianopolis, SC 88020-300, Brazil; ^2^Institute of Biomedical Engineering, Federal University of Santa Catarina, Florianopolis, SC 88040-900, Brazil; ^3^Santa Luzia Medical Laboratory, Florianopolis, SC 88.117-001, Brazil

## Abstract

Most patients with diabetes mellitus are asymptomatic, which leads to delayed and more complex treatment. At the same time, most individuals are routinely subjected to standard clinical laboratory examinations, which create large health datasets over a lifetime. Computer processing has been used to search for health anomalies and predict diseases using clinical examinations. This work studied machine learning models to support the screening of diabetes through routine laboratory tests using data from laboratory tests of 62,496 patients. The classification and regression models used were the K-nearest neighbor, support vector machines, Bayes naïve, random forest models, and artificial neural networks. Glycated hemoglobin, a test used for diabetes diagnosis, was used as the target. Regression models calculated glycated hemoglobin directly and were later classified. The performance of classification computer models has been studied under various subdataset partitions and combinations (e.g., healthy, prediabetic, and diabetes, as well as no healthy and no diabetes). The best single performance was achieved with the artificial neural network model when detecting prediabetes or diabetes. The artificial neural network classification model scored 78.1%, 78.7%, and 78.4% for sensitivity, precision, and F1 scores, respectively, when identifying no healthy group. Other models also had good results, depending on what is desired. Machine learning-based models can predict glycated hemoglobin values from routine laboratory tests and can be used as a screening tool to refer a patient for further testing.

## 1. Introduction

Diabetes mellitus (DM) is a chronic metabolic disorder caused by a deficiency in insulin production or a lack of capability of the cells to use it properly. Over time, DM causes an increase in blood glucose levels, which is known as hyperglycemia. DM also increases the risk of premature death and possible diabetes-associated complications, such as heart attack, stroke, kidney failure, and vision loss [1]. Most DM patients are asymptomatic and do not undergo a DM test, leading to a delayed diagnosis. Late DM identification leads to complex treatment and poor outcomes. It is estimated that DM has an impact of $760 billion costs, accounting for 11.3% of 20–79-year-old deaths worldwide. Early diagnosis is imperative to mitigate diabetes complications and deaths and reduce treatment costs [2].

Currently, DM is diagnosed via analysis of laboratory tests, such as those handling glucose (i.e., fast plasma glucose) and glycated hemoglobin (HbA1c). HbA1c is considered the gold standard for screening and diagnosing diabetes due to its international standardization, lower susceptibility to biological variability, not being affected by acute stress, and no need for fasting [3, 4]. However, FPG exams are still widely used, being often the main diagnostic method. Even though it may present changes in glucose values, leading to erroneous interpretations[3, 5]. For the Hba1c test, the individual is considered healthy if the value is equal to or less than 5.6%, considered prediabetic if the value is between 5.6% and 6.5%, and considered diabetic if the value is equal to or greater than 6.5%. For the FPG test, the individual is considered healthy if the value is equal to or less than 99 mg/dl, considered prediabetic if the value is between 99 mg/dl and 126 mg/dl, and considered diabetic if the value is equal to or greater at 126 mg/dl [1].

Computer processing has been used to identify diseases based on clinical data processing [6–10]. Extracting knowledge from data to support experts in decision-making is a trend in the new generation of smart health systems [11, 12]. Computer methods such as data mining and machine learning can improve diagnosis alongside patient data. Several studies have been using laboratory tests and machine learning techniques to search for new results in recent years. In the case of diabetes mellitus, the search for a diagnosis has been the target of predictive medicine. Many studies have used artificial intelligence to predict a diagnosis or a future propensity to develop the disease. In general, in addition to laboratory tests, these studies make use of clinical data, patient history, imaging tests, and medical diagnoses [13–21], none of which used only laboratory tests. Oleg [14], for example, in addition to laboratory tests, also used data on retinopathy or nephropathy.

Similarly, Hang [16], Wu [19], and Hische [22] also made use of other clinical data in the search for a diagnosis of diabetes. Some studies, such as Ravaut [17], Bernardini [21], and Le [23], aim to determine whether a patient is likely to develop the disease in the future, which is relevant as part of a process in predictive medicine. Other authors [24–28] have used data from noninvasive tests, such as photoplethysmography (PPG) and electrocardiogram (ECG), with the main motivation being the screening and monitoring of blood glucose for patients already diagnosed.

The use of laboratory tests and machine learning to search for new results has been extensively explored in recent years [8, 14, 20, 29–33]. In particular, we draw attention to the work of Park [34], who performed the prediction of several diseases using laboratory tests, but not including DM.

This work has its focus on the use of routine laboratory tests. Once the blood sample has already been collected and the patient's tests performed, the possibility of predicting new information is of great relevance for the diagnostic process of medical laboratories. We do not use any other type of data, enabling the automation of analysis and medical laboratories' diagnostic processes. Discovering information can generate alerts for things not observed, thus proposing complementary exams for an early diagnosis of still unknown pathologies.

For example, in the diagnosis of diabetes mellitus, although the HbA1c test is recommended, the FPG test is the most used. However, this test may present variations and inconsistencies [3, 35], generating false-negative results. It is not uncommon for discrepancies in the result of the diagnosis of DM performed with the FPG test compared to the HbA1c test. In this way, it is crucial to predict possible DM diagnoses and recommend complementary exams to prevent an asymptomatic patient from being left without proper and timely treatment. In this case, the prediction of HbA1c is a possibility to confirm the diagnosis given by the FPG test, and in discrepant cases, it may propose performing the HbA1c test with the blood sample already available. This approach would avoid false-negative results saving time and costs with further exams and treatments.

The possibility of automatically using data from laboratory tests to search for new patient information is of great relevance. This methodology can directly impact the analysis processes of laboratory tests outcome, suggesting complementary and more complex tests in the screening for new pathologies and counter-proof for false-negative cases. In most cases, the blood sample already collected can be used, saving time and costs. Thus, this methodology presents itself as an innovation to performing tests and diagnoses in medical laboratories.

We propose a machine learning-based approach that use existing laboratory data to screen DM based on predicting HbA1c and classifying subjects based on the most frequently performed laboratory examinations: hemograms, creatinine, and fasting plasma glucose. Using these data may enable earlier prediction of HbA1c levels in DM while evaluating routine and straightforward laboratory testing. In this way, the proposed approach can help detect DM by directing the patient to complementary exams. Thus, this work sought to explore and evaluate different machine learning models and dataset configurations to identify the best ways to support the DM diagnosis based on routine laboratory testing.

## 2. Materials and Methods

**Figure 1:**
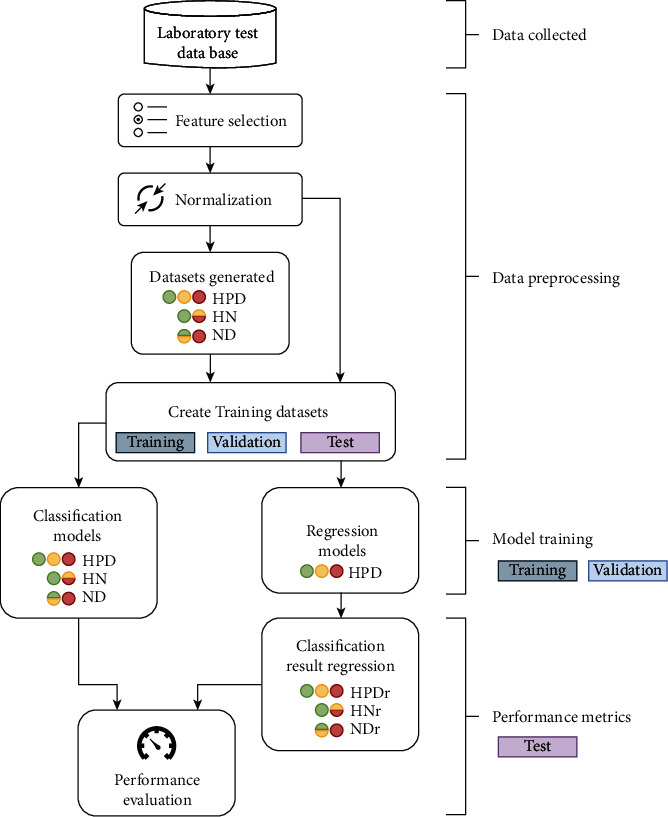
A proposed pipeline for studying the HbA1c classification. There are four main steps: data collection, data preprocessing, model training, and performance evaluation. Three datasets were created: HPD (H = healthy; P = prediabetes; D = diabetes), HN (H = healthy; N = no healthy [N = P + D]), and ND (N = no diabetes [N = H + P]; D = diabetes). Datasets with the suffix *r* refer to the regression models with later classification.

We used a four-step framework to study HbA1c classification models and predictions. The four steps are (1) data collection, (2) data preprocessing, (3) model training, and (4) performance evaluation. The results are shown in Figure 1.

### 2.1. Data Collection

We used a database of laboratory examinations from a clinical analysis laboratory in Florianopolis, Brazil. The dataset included 62,496 patients grouped according to HbA1c values. The dataset included 19–99-year-old adults, 47.60% healthy, 37.95% prediabetes, and 14.45% diabetes. The mean age was 56.7 years (SD = 16.2), and individuals were 43.4% male, and 56.6% were female. The average age was 56.2 years (SD = 15.8) among men and 57.1 years (SD = 16.6) among women. The dataset was randomly split into an 80:20 basis for training and testing. The Federal University of Santa Catarina Ethical Committee approved this study under registration number CAE 02203918.0.0000.0121.

### 2.2. Data Preprocessing

Preprocessing is one of the most important steps in using machine learning techniques. We used a factor analysis technique to select the most relevant examinations for HbA1c levels. Missing data (i.e., any single missing exam) were excluded. No data were inputted.  Table 1 shows the selected features and the target variable HbA1c. The selected features were normalized to a mean of zero and a standard deviation of 1. The classification of HbA1c provides DM diagnoses. There are three HbA1c categories: healthy if HbA1c is <5.7% (39 mmol/mol), prediabetes if HbA1c is between 5.7% and 6.4% (46 mmol/mol), and diabetes if this value is ≥6.5% (48 mmol/mol) [36].

**Figure 2:**
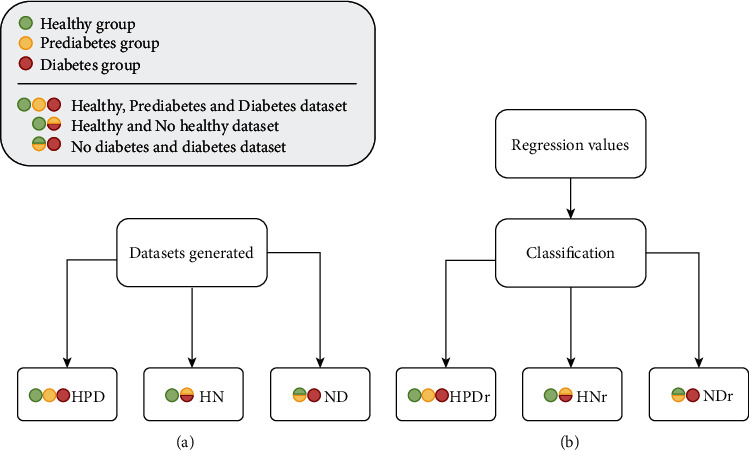
Datasets used with the models. (a) The three subdatasets used with the classification models: healthy, prediabetes, and diabetes (HPD); healthy and no healthy (HN); and no diabetes and diabetes (ND). (b) The three subdatasets used with the regression models (i.e., classification after regression): healthy, prediabetes, and diabetes (HPDr); healthy and no healthy (HNr), being no healthy formed by prediabetes and diabetes; and no diabetes and diabetes (NDr), being no diabetes formed by healthy and prediabetes.

The dataset was arranged into three distinct subdatasets, “HPD,” “HN,” and “ND,” using the classification models (see Figure 2(a)). The first subdataset, “HPD,” describes individuals based on HbA1c; thus, there are three categories: healthy (“H”), prediabetes (“P”), and diabetes (“D”). The second subdataset, “HN,” describes individuals as healthy (“H”) and no healthy (“N”), where “N” is the prediabetes and diabetes (N = P + D). The third subdataset “ND” describes individuals as no diabetes (“N”) and diabetes (“D”), where “N” is the healthy and prediabetes (N = H + P). The dataset was also arranged into three subdatasets using the regression models' classification (see Figure 2(b)) that subdatasets acronym follows the pattern of the subdatasets generated for using with the classification models with the addition of “r” suffix, i.e., “HPDr,” “HNr,” and “NDr.”

### 2.3. Model Training

We trained five classification models and five regression models. The target of the classification model was an ordinary variable HbA1c, and the target for the regression models was a continuous variable HbA1c. The regression model HbA1c output was classified based on DM classification. We used several models with different approaches and complexities, ranging from simple K-nearest neighbors to complex ANNs. The Python package Scikit-learn [37] was used to implement the models. For validation, 30% of the training part of the dataset was used. The training and validation approaches were used for hyperparameter tuning. The adjustment of hyperparameters for the models was performed using Bayesian optimization (BO) with a Gaussian process (GP) [38].

The classification was performed using K-nearest neighbors (KNN), support vector machine (SVM), naïve Bayes (NB), random forest (RF), and artificial neural networks (ANN). The regression was studied using these methods as regressors, i.e., K-nearest neighbor regressor (KNNr), support vector machine regressor (SVMr), naïve Bayes regressor (NBr), random forest regressor (RFr), and artificial neural networks regressor (ANNr). The following configuration was used:

- KNN and KNNr model hyperparameters were set to “8 neighbors,” “uniform weights,” and “ball tree algorithm”
- SVM model hyperparameters was set “0,8 C,” “RBF kernel,” “3 degree,” “true shrinking,” “true probability,” “decision function shape over,” and “1000 cache”
- SVMr model hyperparameters were set to “1 C,” “epsilon insensitive loss,” “0.1 epsilon” and tolerance of 1e5. “decision function shape over,” and “1000 cache”
- NB and NBr were set to default
- RF and RFr was set to “gini criterion,” “5 max depth,” and “50 estimators”
- ANN and ANNr were set to “2 layers with 50 neurons,” “adam solver,” “adaptative learning rate,” and “relu activation”

### 2.4. Performance Metrics

The test part of the dataset is used to evaluate the results. We used the mean squared error (MSE) to assess regression performance using equation (1), where the *n* represents the number of samples, *y*_*i*_ represents the original value of all *i* samples, and ${\hat{\text{y}}}_{i}$ represents the predicted values of all *i* samples [39].

(1) $$\begin{array}{c} \text{MSE}=\frac{1\;}{n}\sum {\left(y_{i}-{\overset{\wedge }{y}}_{i}\right)}^{2}. \end{array}$$

HN and ND were compared to HNr and NDr to evaluate whether the machine learning models. Prediabetes is the midstage between healthy (no diabetes) and diabetes and has a narrow HbA1c value range; this relationship might negatively influence model performance. Five metrics were used to study the models: sensitivity (SN), specificity (SP), precision (PR), negative precision (NPR), and F1 score (F1), as in equations ((2)–(6)). The true positives (TP) and true negatives (TN) are the number of positive samples in the positive set and the number of identified negative samples in the negative set. The false-positive (FP) and false-negative (FN) values are the numbers of positive samples in the negative set and the number of negative samples identified in the positive set. Sensitivity is the true positive rate, and specificity is the true negative rate. The F1 score is the harmonic mean of precision and sensitivity. The F1 score is the harmonic mean of precision and sensitivity, recommended for use with unbalanced databases, such as the database used in this work. The confusion matrix was used to visualize the performance of the algorithms; the rows represent the predicted class, the columns represent the actual class, and a good model must have a true diagonal near 1 [39].

(2) $$\begin{array}{c} \text{SN}=\frac{\text{TP}}{\text{TP}+\text{FN}}, \end{array}$$

(3) $$\begin{array}{c} \text{SP}=\frac{\text{TN}}{\text{TN}+\text{FP}}, \end{array}$$

(4) $$\begin{array}{c} \text{PR}=\frac{\text{TP}}{\text{TP}+\text{FP}}, \end{array}$$

(5) $$\begin{array}{c} \text{NPR}=\frac{\text{TN}}{\text{TN}+\text{FN}}, \end{array}$$

(6) $$\begin{array}{c} \text{F}1=\frac{2\text{TP}}{2\text{TP}+\text{FN}+\text{FP}}. \end{array}$$

## 3. Results

**Figure 3:**
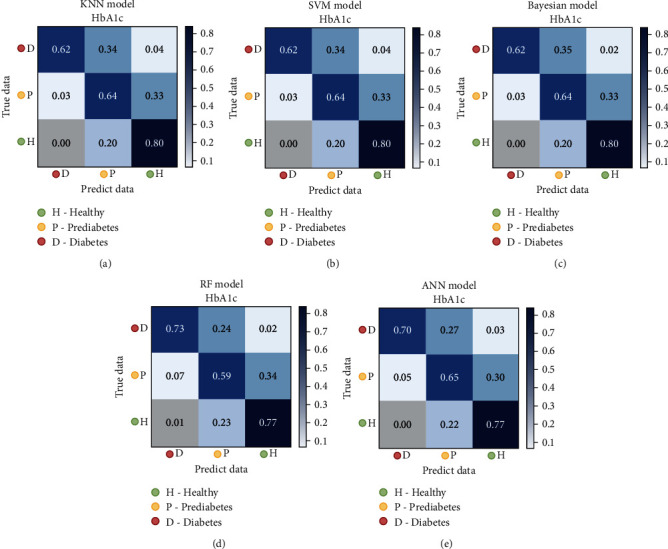
Confusion matrices for the classification models using the HPD dataset: (a) KNN, (b) SVM, (c) NB, (d) RF, and (e) ANN.

Table 2 lists the performance of the classification model for classifying the HPD dataset. The models had different score characteristics. The ANN model has greater sensitivity in identifying people with diabetes, although the precision is not high (84.9%). On the other hand, the KNN model has a lower sensitivity in identifying DM but greater precision within the identified DM.  Figure 3 shows the confusion matrix of the classification models using the HPD dataset. We found that KNN, SVM, and NB behaved approximately equally, while the ANN performed better than the others. All the models have approximately a 30% prediction error for prediabetes, which indicates that this category is primarily fuzzy. In addition, there is a tendency to misclassify prediabetic individuals as healthy than diabetics, which may be due to the dataset characteristics.

**Figure 4:**
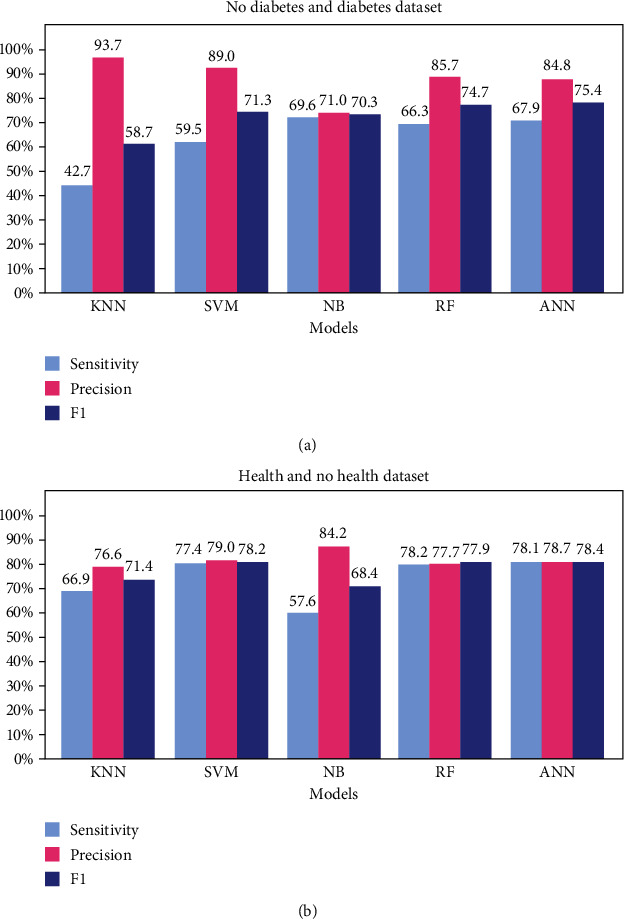
Sensitivity, precision, and F1 score of the classification models for the HN (healthy versus no healthy) and ND (no diabetes versus diabetes) datasets.

The performance of the classification models for the HN and ND datasets is presented in Table 3. The sensitivity, precision, and F1 score are shown as bar plots in Figure 4. The HN models are more regular regarding scores; however, they have approximately 70%–80% precision; thus, some false positives are expected. We observe that the KNN has a sensitivity of only 42.7% using ND; however, it leads to high precision, useful in screening false negatives.

**Figure 5:**
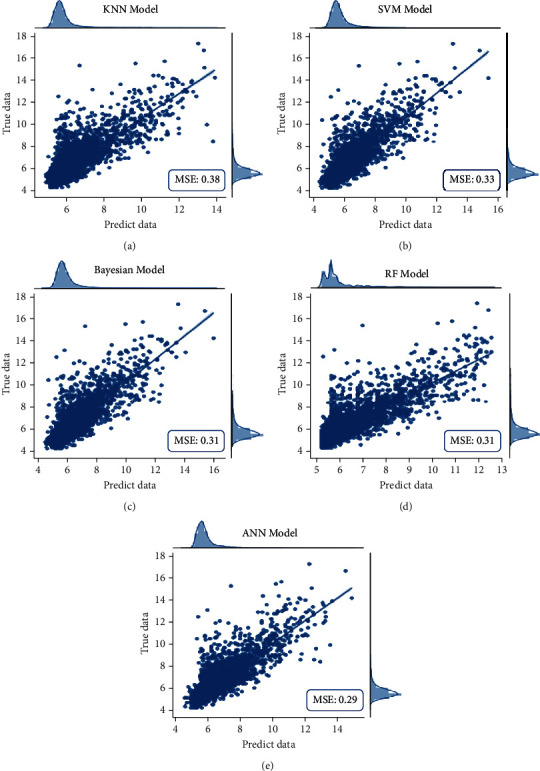
Regression models MSE: (a) KNN, (b) SVM, (c) NB, (d) RF, and (e) ANN.

**Figure 6:**
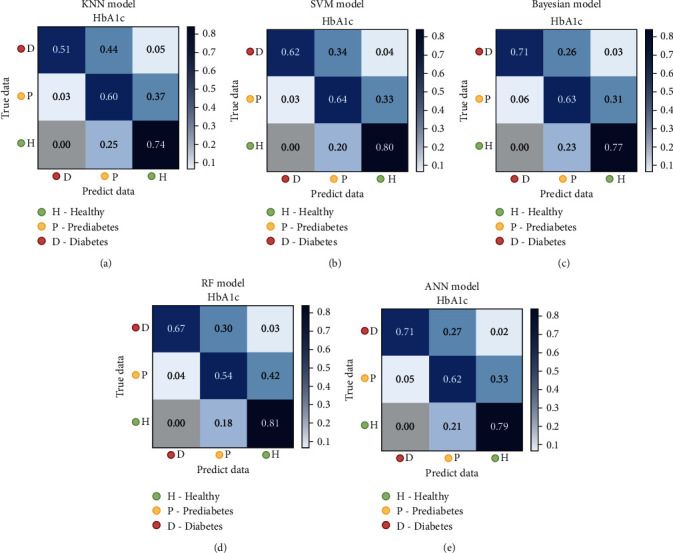
Confusion matrices for the regression models using healthy, prediabetes, and diabetes (HPDr) dataset: (a) KNNr, (b) SVMr, (c) NBr, (d) RFr, and (e) ANNr.

Figure 5 shows the regression model errors (MSE results). The regression line is shown. Data points are clustered in the regression line, which is indicative of the excellent performance of the models. The average MSE of the five models is 0.32, with the best performance achieved by the ANN model (0.29) and the worst, 0.38, by KNN.  Table 4 shows the performance of the predicted values of the regression, arranged as HPDr.  Figure 6 shows the relative confusion matrix. The regression models make it possible to observe the misclassification tendency of prediabetics as healthy compared to individuals with diabetes. This tendency was also observed when using the classification models (see Figure 3). Thus, this tendency may be a characteristic of the data and not an imbalance in the database.

**Figure 7:**
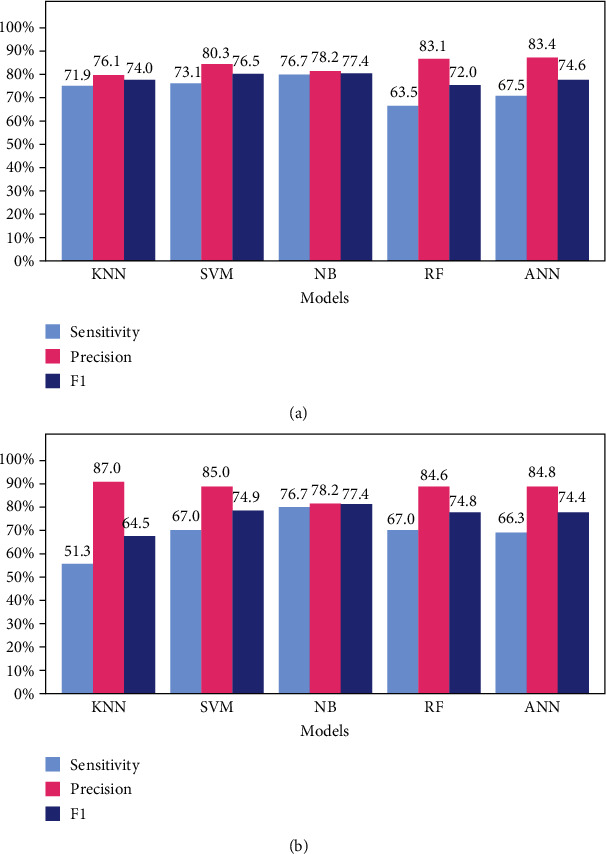
Sensitivity, precision, and F1 score of the regression models for the HN (healthy versus no healthy) and ND (no diabetes versus diabetes) datasets.

The performance of the regression models using HNr and NDr is presented in Table 4. The sensitivity, precision, and F1 score are also shown as bar plots in Figure 7. The results presented by the classification and regression models were similar when analyzing the same “type” of machine learning model. This characteristic can be observed in the three tested datasets (HPD, HN, and ND). Some of the tested machine learning models showed a slight improvement in performance with classification after regression.

## 4. Discussion

We studied a machine learning approach to detect DM using data from the most frequently performed clinical laboratory examinations. Glycated hemoglobin was used as a model target. Clinical laboratory data are often available because they are typically generated from routine blood tests. We demonstrated that machine learning could assist in the detection of DM. This system can be implemented at a minimal cost, as data are already available on computer databases from routine examinations. The proposed approach alone is not recommended for diagnostic purposes. We recommend using the system to generate an alert and recommend a specific DM examination. Thus, the models would improve DM investigation processes, as patients diagnosed with prediabetes or diabetes could be referred for further analysis, which is compatible with intelligent health systems [40]. If a patient laboratory log shows diabetes probability, i.e., a “diabetes-like” pattern, the system can recommend further diabetes examination. The patients with “diabetes patterns” should be guided to traditional examinations and procedures.

HbA1c strongly correlates with the average glucose [41], being more stable and recommended for diabetes diagnoses [42]. Thus, during the FPG analysis process, the system will be able to predict HbA1c values over different arrangements of datasets, looking for some kind of discrepancy in relation to the exam performed. If there is a difference between the results, a new FPG test or a supplementary HbA1c test may be recommended in order to obtain a counter-proof of the result. As it is a computational method, there is no interruption in traditional procedures. The system can collaborate synergistically with the current procedure and may collaborate to detect DM earlier.

Currently, some studies have used laboratory tests to predict new results and support the diagnosis of diseases that are not the target of the test, as in Park's study [34], where several diseases are predicted. The most recent reports were [43–46], which studied the prediction of the RT-PCR test. However, several studies have used other types of data and machine learning techniques to assist DM prediction. In a study by Zheng et al. [47], the authors obtained 100% sensitivity and precision above 90% in several models while using a dataset of 300 samples and used several categories of features, including self-reporting notes and medication. Oliveira et al. [48] obtained 68% sensitivity and 68% specificity after using a smaller dataset and categorical features obtained through interviews. Lai et al. [16] obtained 71.6% sensitivity and 73.4% specificity with a dataset of 13,309 samples using laboratory and clinical features. The results obtained in this study cannot be directly compared to those of the studies mentioned above because the methodologies and features (i.e., input parameters and hyperparameters) differ. This study used only quantitative data from routine laboratory tests to train different classification and regression models, as well as different dataset arrangements, having an exploratory character.

A confusion matrix was chosen for the evaluation of the overall model. The confusion matrix is particularly useful when working with unbalanced data. The values of the main diagonal of the confusion matrix make up the accuracy, which is not a good evaluation metric in classification models with unbalanced datasets. In these cases, the F1 score is the most recommended evaluation metric. This F1 score represents a consonant mean between sensitivity and precision and is a simple way to evaluate models with unbalanced databases. However, the most appropriate metric to evaluate a classification model in searching for a target is the joint analysis of sensitivity and precision. For instance, high-sensitivity models are better for target identification (e.g., RF model for HN dataset, Table 3). Therefore, prioritizing models with high precision (e.g., KNN model for ND dataset, Table 3) will provide greater certainty in the results.

When using the classification models (see Table 2), we found that the ANN model had the highest sensitivity in identifying DM (66.2%), with a precision of 84.9%. The same occurred in identifying patients with prediabetes, where the ANN model had the best sensitivity (67.9%). The KNN model, on the other hand, obtained the highest precision in the identification of DM (89.1%), despite the low sensitivity (47.0%). For the identification of healthy individuals, the NB model had the highest sensitivity (79.4%), followed by the SVM and RF models (79.2% for both). The highest precision was for the ANN model (76.9%), followed by the SVM and RF models (74.2% for both). Regarding the F1 score, we found that the ANN model had the highest results for all classes (i.e., 74.4% for diabetes, 64.9% for prediabetes, and 76.7% for healthy).

When using the regression models, we verified the capacity of the models in predicting HbA1c, as shown in the scatter plot in Figure 5. ANNr yielded the best result, with an MSE of 0.29. However, the graph shows that all models were able to predict HbA1c. Subsequently, the predicted value of HbA1c was classified as DM status according to [36], which may lead to some classification errors when HbA1c values are close to the transition limits between the different classes. This fuzzy range in the classification of regression values is proportional to the mean absolute error (MAS) of approximately 0.33 for all tested models.

Comparing the values in Tables 4 and 5, we observe a certain similarity of the classification results after regression with the results of the classification models. This similarity was also observed by examining the confusion matrix (Figures 3 and 6). Among the different models and datasets, a slight variation in the results was observed; in some cases, the classification of regression values was better than that of the classification models. Figures 3 and 6 show that all models misclassified (by more than 30%) prediabetes cases as healthy cases. Thus, according to the classification of the models, patients with prediabetes are more “similar” to healthy individuals than patients with DM. We can further analyze the prediabetic classification characteristics using the HN and ND datasets.

In Table 3, we observe that the ANN model performed better using the HN dataset (where prediabetes and DM are in the same class), with a sensitivity of 78.1% and precision of 78.7%. This arrangement is interesting in the search for unhealthy individuals and can be used in general to screen patients who already have or are on the way to developing the disease. However, regarding the sensitivity and precision using the ND dataset (where healthy and prediabetic individuals are in the same class), we observed variations in the performance of the models. All models have lower sensitivity but higher precision values than the HN dataset. According to the F1 score, the regression model that classified better patients with diabetes and prediabetes was SVMr, reaching 74.9% and 61.1%, respectively. The model with the best performance in classifying healthy patients was ANNr (77.2%). The greater precision of the models in the ND dataset reinforces the idea that prediabetes patients are more similar to healthy individuals than they are to patients with diabetes.

Depending on the objective, a dataset arrangement of HN or ND can be used. For instance, precision is more important than sensitivity when screening for false negatives, which would lead us to use the ND arrangement. Only the results with a negative diagnosis would be analyzed in this case. Even if the system is not very sensitive, it must have high precision. Thus, even if a few cases of false negatives are identified, we will be more confident that these cases are real false negatives. In this sense, we draw attention to the KNN classification model. Using this model in the search for false negatives, even if it only identifies half of the occurrences, we will be 94% sure that these tests are false negatives.

In the search for the correct classification of diabetes or no healthy patients, we understand that the idea is to have high sensitivity and precise models, which means fewer false positives. We demonstrate that machine learning can detect DM using data from laboratory examinations performed most frequently. The model achieved better results as the sensitivity increased; however, sensitivity was less important than precision.

The artificial neural network classification model scored 78.1%, 78.7%, and 78.4% for sensitivity, precision, and F1 scores, respectively, when identifying no healthy individuals (i.e., individuals with prediabetes or diabetes). Thus, we believe that this approach exhibits the best overall performance. We observed that all tested models had difficulty classifying the prediabetes group; thus, the dataset configuration improved detection. This model may use existing laboratory examinations of patients to recommend further and specific DM follow-up. Thus, these results could support the screening of DM using machine learning algorithms and available clinical information.

## 5. Conclusions

Patients with DM may be asymptomatic and go unnoticed in diagnoses based only on FPG exams. These exams can vary and be susceptible to nonstandard methodologies, patient adherence and preparation prior to the exam, and medications in use.

The possibility for a computer system to automatically find hidden information in laboratory test data is highly advantageous to the diagnostic process in medical laboratories. These systems could perform patient screening to discover early diseases, generate alerts, and recommend complementary exams to counter-proof possible problems with false negatives. These tests could be performed with the patient's blood sample, usually already available.

This work demonstrates that machine learning models can aid in DM screening using data from routinely performed laboratory tests, including blood counts, providing evidence to refer a patient for further testing (e.g., HbA1c). The proposed system can operate in conjunction with traditional methods and not interrupt the normal flow process of exams.

Different dataset arrangements and prediction models can be used depending on the purpose or application of this approach. For example, to perform a screening in the search for individuals with DM, one option would be to use the ANN model with the HN dataset, and this is because it presents greater sensitivity and maintains good precision.

If the objective is to find false negatives in an FPG exam, we could use the KNN classification model with the ND dataset. Despite having low sensitivity, this arrangement presented the highest precision, thus reinforcing the certainty in the results found.

The next step in this study is the improvement of methods that help discover false negatives with the FPG exam. Because it is the most performed test in the search for a diagnosis of DM and presents possible variations, this process may inhibit the early treatment of asymptomatic patients.

Early detection of DM is advantageous for the health system and patients as it reuses existing laboratory information. Detecting DM earlier can improve the quality of life and reduce treatment complexity, costs, and late complications.

## Figures and Tables

**Table 1:** Description of the preprocessed dataset.

Attribute	Description	Min.	Max.	Mean	SD
*HbA1c*	*Glycated hemoglobin (%, percent)*	*3.20*	*18.30*	*5.92*	*1.01*
Age	Age of the patient (year)	20	99	56.75	16.25
CR	Creatinine (mg/dl, milligram/decaliter)	0.29	18.23	0.92	0.37
FPG	Fast plasma glucose (mg/dl, milligram/decaliter)	38.00	626.00	102.05	29.99
Baso%	Percent bashophils (%, percent)	0.10	3.70	0.75	0.32
MCHC	Mean corpuscular hemoglobin concentration (g/dl, gram/decaliter)	24.90	38.90	33.44	0.91
MCH	Mean corpuscular hemoglobin (pg, picogram)	14.90	42.30	29.70	1.47
HT	Erythrocytes (%, percent)	16.30	60.30	41.20	3.87
LC	Leukocytes (unit per mm2)	720.00	27710	6504.8	1819.6
Linfo%	Percent lymphocytes (unit per mm2)	4.10	85.30	34.14	8.21
Mono%	Percent monocytes (unit per mm2)	0.70	34.30	6.13	1.53
MPV	Mean platelet volume (%, percent)	5.60	19.20	8.62	1.07
PLT	Absolute platelet count (unit per mm2)	4.00	796.0	251.9	67.46
RDW	Red cell distribution width (%, percent)	10.80	26.80	13.60	0.95
SEG%	Percent segmented neutrophils (unit per mm2)	6.70	93.90	55.73	8.72
MVC	Mean corpuscular volume (ft, femtoliter)	52.20	127.2	88.81	4.89

**Table 2:** Performance of the studied classification models using healthy, prediabetes, and diabetes (HPD) dataset.

Class	KNN	SVM	NB	RF	ANN
*Sensitivity (SN %)*					
Diabetes	47.0	62.4	63.1	62.4	66.2
Prediabetes	53.4	63.7	56.3	63.7	67.9
Healthy	78.7	79.2	79.4	79.2	76.6
*Specificity (SP %)*					
Diabetes	99.0	98.4	95.9	98.4	98.0
Prediabetes	74.2	76.5	77.8	76.5	75.0
Healthy	64.6	75.0	72.6	75.9	79.1
*Precision (PR %)*					
Diabetes	89.1	86.9	72.3	86.9	84.9
Prediabetes	55.7	62.2	50.6	62.2	62.2
Healthy	66.9	74.2	72.5	74.2	76.9
*Negative precision (NPR %)*					
Diabetes	91.6	93.8	93.8	93.8	94.4
Prediabetes	72.5	77.7	74.6	77.7	79.4
Healthy	76.9	79.8	79.5	79.8	78.8
*F1 score (F1 %)*					
Diabetes	61.8	72.6	67.4	72.6	74.4
Prediabetes	54.5	62.6	58.3	62.9	64.9
Healthy	72.3	76.6	75.8	76.6	76.7

**Table 3:** Performance of the classification models using healthy and no healthy (HN) and no diabetes and diabetes (ND) datasets. Percentual (%) values of sensitivity (SN), specificity (SP), precision (PR), negative precision (NPR), and F1 score (F1).

	Healthy and no healthy (%)					No diabetes and diabetes (%)				
	KNN	SVM	NB	RF	ANN	KNN	SVM	NB	RF	ANN
SN	55.9	77.3	57.6	78.2	*78.1*	*42.7*	59.9	69.6	66.3	67.9
SP	77.5	77.5	88.1	75.3	76.6	99.5	98.7	95.1	98.1	97.9
PR	76.6	79.0	84.2	77.7	*78.7*	*93.7*	89.0	71.0	85.7	84.8
NPV	68.1	75.6	65.4	75.8	76.1	91.0	93.4	94.8	94.4	94.7
F1	71.4	78.2	68.4	77.9	*78.4*	58.7	71.3	70.3	74.7	75.4

**Table 4:** Performance of the studied regression models using healthy, prediabetes, and diabetes (HPDr) dataset.

Class	KNNr	SVMr	NBr	RFr	ANNr
*Sensitivity (SN %)*					
Diabetes	51.3	67.0	63.1	68.0	66.3
Prediabetes	60.7	59.9	56.3	47.1	51.7
Healthy	75.2	80.2	79.4	85.8	85.3
*Specificity (SP %)*					
Diabetes	98.7	98.0	95.9	97.9	98.0
Prediabetes	71.2	78.2	77.8	82.9	81.7
Healthy	71.9	73.1	72.6	63.5	67.5
*Precision (PR %)*					
Diabetes	87.0	85.0	72.3	84.6	84.8
Prediabetes	56.0	62.4	60.6	62.5	63.1
Healthy	70.9	73.1	72.5	68.2	70.5
*Negative precision (NPR %)*					
Diabetes	92.2	94.5	93.8	94.5	94.4
Prediabetes	74.9	76.3	74.6	72.1	73.6
Healthy	76.1	80.3	79.5	83.1	83.4
*F1 score (F1 %)*					
Diabetes	64.5	74.9	67.4	74.8	73.4
Prediabetes	58.3	61.1	58.3	53.7	56.8
Healthy	73.0	76.5	75.8	73.0	77.2

**Table 5:** Performance of the regression models using healthy and no healthy (HN) and no diabetes and diabetes (ND) datasets. Percentual (%) values of sensitivity (SN), specificity (SP), precision (PR), negative precision (NPR), and F1 score (F1).

	Healthy and no healthy (%)					No diabetes and diabetes (%)					
	KNN	SVM	NB	RF	ANN	KNN	SVM	NB	RF	ANN
SN	71.9	73.1	76.7	63.5	67.5	51.3	67.0	76.7	67.0	66.3
SP	86.6	80.9	77.1	85.0	75.2	99.2	98.0	77.1	98.0	97.5
PR	76.1	80.3	78.2	83.1	83.4	87.0	85.0	78.2	84.6	84.8
NPV	79.7	72.3	74.3	68.4	75.4	94.3	94.0	74.3	93.9	94.6
F1	74.0	76.5	77.4	72.0	74.6	64.5	74.9	77.4	74.8	74.4

## Data Availability

The data used to support the findings of this study were supplied by Santa Luzia Medical Laboratory under license and cannot be made freely available. Reasonable requests for accessing these data should be made to the corresponding author.

## References

[1] World Health Organisation (2016). Global report on diabetes.

[2] Federation I. D. (2019). *International Diabetes Federation*.

[3] Sacks D. B. (2011). A1C versus glucose testing: a comparison. *Diabetes Care*.

[4] Nathan D. M., Balkau B., Bonora E. (2009). International expert committee report on the role of the A1C assay in the diagnosis of diabetes. *Diabetes Care*.

[5] National Institute of Diabetes and Digestive and Kidney Diseases (2021). The A1C Test & Diabetes|NIDDK. https://www.niddk.nih.gov/health-information/diagnostic-tests/a1c-test.

[6] Chen M., Hao Y., Hwang K., Wang L., Wang L. (2017). Disease prediction by machine learning over big data from healthcare communities. *IEEE Access*.

[7] Hossain M. E., Khan A., Moni M. A., Uddin S. (2021). Use of electronic health data for disease prediction: a comprehensive literature review. *Transactions On Computational Biology And Bioinformatics*.

[8] Luo Y., Szolovits P., Dighe A. S., Baron J. M. (2016). Using machine learning to predict laboratory test results. *American Journal of Clinical Pathology*.

[9] Baron J. M., Dighe A. S. (2014). The role of informatics and decision support in utilization management. *Clinica Chimica Acta*.

[10] Ma H., Xu C. F., Shen Z., Yu C. H., Li Y. M. (2018). Application of machine learning techniques for clinical predictive modeling: a cross-sectional study on nonalcoholic fatty liver disease in China. *BioMed Research International*.

[11] Fernández-Llatas C., García-Gómez J. M. (2015). *Data Mining in Clinical Medicine*.

[12] Peek N., Combi C., Marin R., Bellazzi R. (2015). Thirty years of artificial intelligence in medicine (AIME) conferences: a review of research themes. *Artificial Intelligence in Medicine*.

[13] De Silva K., Mathews N., Teede H. (2021). Clinical notes as prognostic markers of mortality associated with diabetes mellitus following critical care: a retrospective cohort analysis using machine learning and unstructured big data. *Computers in Biology and Medicine*.

[14] Metsker O., Magoev K., Yakovlev A. (2020). Identification of risk factors for patients with diabetes: diabetic polyneuropathy case study. *BMC Medical Informatics and Decision Making*.

[15] Du Y., Fang Z., Jiao J. (2021). Application of ultrasound-based radiomics technology in fetal-lung-texture analysis in pregnancies complicated by gestational diabetes and/or pre-eclampsia. *Ultrasound in Obstetrics & Gynecology*.

[16] Lai H., Huang H., Keshavjee K., Guergachi A., Gao X. (2019). Predictive models for diabetes mellitus using machine learning techniques. *BMC Endocrine Disorders*.

[17] Ravaut M., Harish V., Sadeghi H. (2021). Development and validation of a machine learning model using administrative health data to predict onset of type 2 diabetes. *JAMA Network Open*.

[18] Camargo J. L., Gross J. L. (2004). Glycohemoglobin (GHb): clinical and analytical aspects. *Arquivos Brasileiros de Endocrinologia & Metabologia*.

[19] Wu Y.-T., Zhang C. J., Mol B. W. (2021). Early prediction of gestational diabetes mellitus in the Chinese population via advanced machine learning. *The Journal of Clinical Endocrinology and Metabolism*.

[20] Yu L., Li L., Bernstam E., Jiang X. (2020). A deep learning solution to recommend laboratory reduction strategies in ICU. *International Journal of Medical Informatics*.

[21] Bernardini M., Morettini M., Romeo L., Frontoni E., Burattini L. (2019). TyG-er: An ensemble Regression Forest approach for identification of clinical factors related to insulin resistance condition using Electronic Health Records. *Computers in Biology and Medicine*.

[22] Hische M., Luis-Dominguez O., Pfeiffer A. F. H., Schwarz P. E., Selbig J., Spranger J. (2010). Decision trees as a simple-to-use and reliable tool to identify individuals with impaired glucose metabolism or type 2 diabetes mellitus. *European Journal of Endocrinology*.

[23] Le T. M., Vo T. M., Pham T. N., Dao S. V. T. (2021). A novel wrapper-based feature selection for early diabetes prediction enhanced with a metaheuristic. *IEEE Access*.

[24] Zhang G., Mei Z., Zhang Y. (2020). A noninvasive blood glucose monitoring system based on smartphone PPG signal processing and machine learning. *Transactions on Industrial Informatics*.

[25] Nirala N., Periyasamy R., Singh B. K., Kumar A. (2019). Detection of type-2 diabetes using characteristics of toe photoplethysmogram by applying support vector machine. *Biocybernetics and Biomedical Engineering*.

[26] Keikhosravi A., Aghajani H., Zahedi E. (2013). Discrimination of bilateral finger photoplethysmogram responses to reactive hyperemia in diabetic and healthy subjects using a differential vascular model framework. *Physiological Measurement*.

[27] Prabha A., Yadav J., Rani A., Singh V. (2021). Design of intelligent diabetes mellitus detection system using hybrid feature selection based XGBoost classifier. *Computers in Biology and Medicine*.

[28] Avram R., Tison G., Kuhar P. (2019). Predicting diabetes from photoplethysmography using deep learning. *Journal of the American College of Cardiology*.

[29] Schneider J. L., Layefsky E., Udaltsova N., Levin T. R., Corley D. A. (2020). Validation of an algorithm to identify patients at risk for colorectal cancer based on laboratory test and demographic data in diverse, community-based population. *Clinical Gastroenterology and Hepatology*.

[30] de Souza A. A., Almeida D. C. ., Barcelos T. S. (2021). Simple hemogram to support the decision-making of COVID-19 diagnosis using clusters analysis with self-organizing maps neural network. *Soft Computing*.

[31] Yang H. S., Hou Y., Vasovic L. V. (2020). Routine laboratory blood tests predict SARS-CoV-2 infection using machine learning. *Clinical Chemistry*.

[32] Richardson A. M., Lidbury B. A. (2017). Enhancement of hepatitis virus immunoassay outcome predictions in imbalanced routine pathology data by data balancing and feature selection before the application of support vector machines. *BMC Medical Informatics and Decision Making*.

[33] Tamune H., Ukita J., Hamamoto Y., Tanaka H., Narushima K., Yamamoto N. (2020). Efficient prediction of vitamin B deficiencies via machine-learning using routine blood test results in patients with intense psychiatric episode. *Frontiers in Psychiatry*.

[34] Park D. J., Park M. W., Lee H., Kim Y. J., Kim Y., Park Y. H. (2021). Development of machine learning model for diagnostic disease prediction based on laboratory tests. *Scientific Reports*.

[35] Troisi R. J., Cowie C. C., Harris M. I. (2000). Diurnal Variation in Fasting Plasma Glucose. *JAMA*.

[36] Shubrook J., Butts A., Chamberlain J. J. (2017). Standards of medical care in diabetes—2017: summary of revisions. *Diabetes Care*.

[37] Pedregosa F., Varoquaux G., Gramfort A. (2011). Scikit-learn: machine learning in python. *Journal of Machine Learning Research*.

[38] Snoek J., Larochelle H., Adams R. P. (2012). Practical Bayesian optimization of machine learning algorithms. *Advances in Neural Information Processing Systems*.

[39] James G., Witten D., Hastie T., Tibshirani R. (2000). An introduction to statistical learning. *Current Medicinal Chemistry*.

[40] Moreira M. W. L., Rodrigues J. J. P. C., Korotaev V., Al-Muhtadi J., Kumar N. (2019). A comprehensive review on smart decision support systems for health care. *IEEE Systems Journal*.

[41] Nathan D. M., Kuenen J., Borg R., Zheng H., Schoenfeld D., Heine R. J. (2008). Translating the A1C assay into estimated average glucose values for the A1C-derived average glucose (ADAG) study group.

[42] Van‘t Riet E., Alssema M., Rijkelijkhuizen J. M., Kostense P. J., Nijpels G., Dekker J. M. (2010). Relationship between A1C and glucose levels in the general Dutch population: the new Hoorn study. *Diabetes Care*.

[43] AlJame M., Imtiaz A., Ahmad I., Mohammed A. (2021). Deep forest model for diagnosing COVID-19 from routine blood tests. *Scientific Reports*.

[44] Rahman T., Al-Ishaq F. A., Al-Mohannadi F. S. (2021). Mortality prediction utilizing blood biomarkers to predict the severity of COVID-19 using machine learning technique. *Diagnostics*.

[45] Myari A., Papapetrou E., Tsaousi C. (2022). Diagnostic value of white blood cell parameters for COVID-19: is there a role for HFLC and IG. *International Journal of Laboratory Hematology*.

[46] Campagner A., Carobene A., Cabitza F. (2021). External validation of machine learning models for COVID-19 detection based on complete blood count. *Health Information Science and Systems*.

[47] Zheng T., Xie W., Xu L. (2017). A machine learning-based framework to identify type 2 diabetes through electronic health records. *International Journal of Medical Informatics*.

[48] Olivera A. R., Roesler V., Iochpe C. (2017). Comparison of machine-learning algorithms to build a predictive model for detecting undiagnosed diabetes - ELSA-Brasil: accuracy study. *São Paulo Medical Journal*.

