# Supplementary figures and images for: Use of Machine Learning and Routine Laboratory Tests for Diabetes Mellitus Screening
Source: Biomed Res Int. 2022 Mar 29;2022:8114049. doi: 10.1155/2022/8114049 (PMC8983182; doi:10.1155/2022/8114049)

# Use of Machine Learning and Routine Laboratory Tests for Diabetes Mellitus Screening

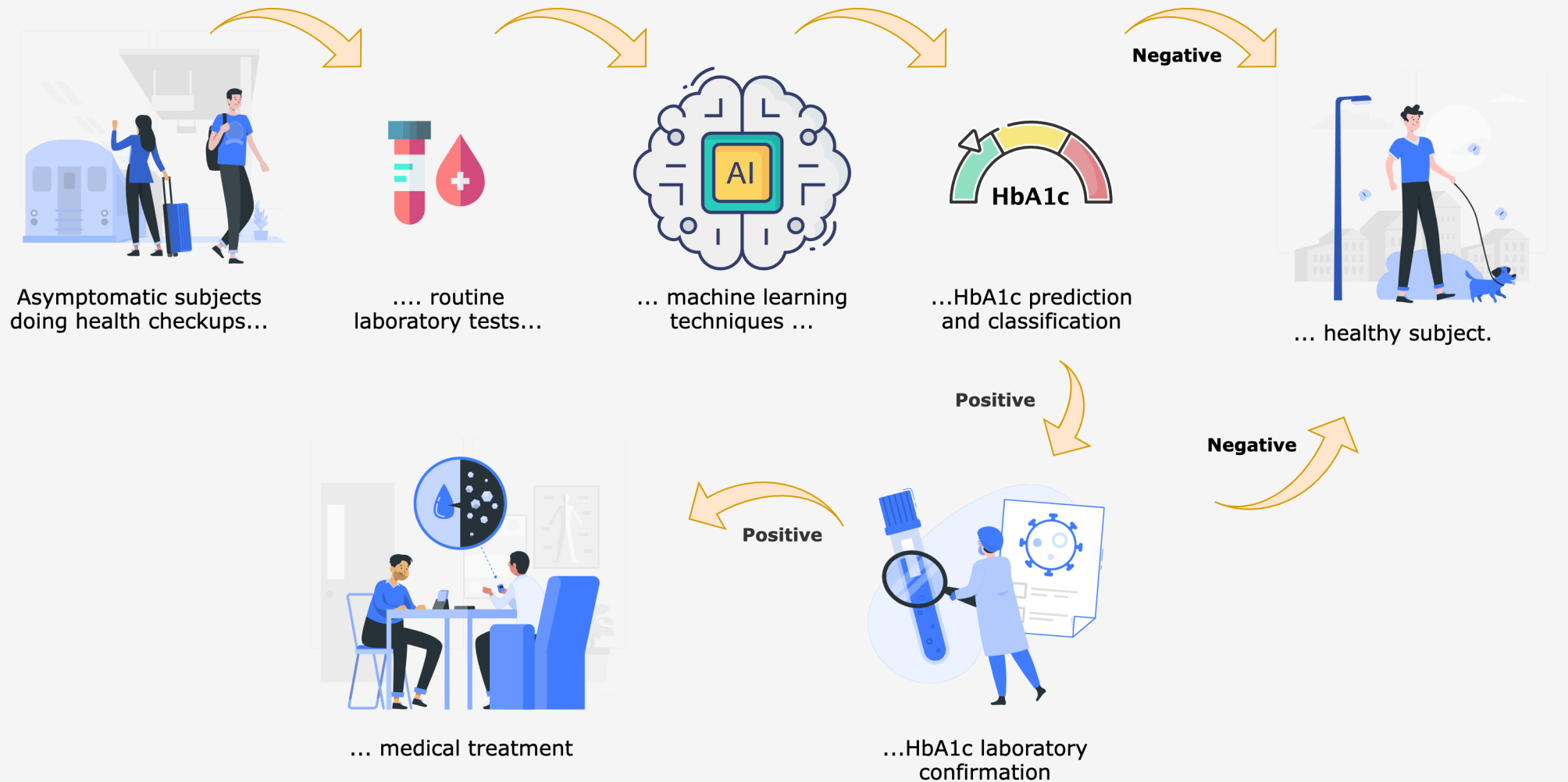

Supplement: Supplementary Materials [file 8114049.f1.pdf]
